# The role of gene regulatory evolution in weapon diversification: Insights from comparative genomics, chromatin profiling, and functional analyses of BMP signaling in horned beetles

**DOI:** 10.1371/journal.pgen.1012338

**Published:** 2026-10-21

**Authors:** Phillip L. Davidson, Isabel G. Manley, Armin P. Moczek

**Affiliations:** 1 Department of Biological Sciences, Mississippi State University, Starkville, Mississippi, United States of America; 2 Department of Biology, Indiana University, Bloomington, Indiana, United States of America; Cornell University, UNITED STATES OF AMERICA

## Abstract

Condition-responsive growth is a common feature of multicellular development, during which organisms integrate genetic and environmental signals to adjust their phenotype. However, the molecular properties shaping condition-responsive diversification remain poorly understood. The beetle genus *Onthophagus* offers promising opportunities to investigate this question, given its diversity of sex- and nutrition-responsive phenotypes, including horns — novel, secondary sexual weapons used in reproductive competition. We compared the gene regulatory properties of horns in two closely related species exhibiting highly specialized sex- and nutrition-biased phenotypes. We found a dynamic *cis*-regulatory landscape in developing horn tissue, shaped by mutational accumulation across species, condition-specific changes in chromatin accessibility within species, and the acquisition of lineage-specific regulatory elements. Many of these putative regulatory regions include binding site motifs of critical developmental regulatory genes, including members of the bone morphogenetic protein signaling pathway. We provide functional evidence that two proteins in this pathway have been co-opted for horn development, influencing the shape and size of this trait across species, sexes, and nutritional states. These findings provide evidence that evolutionarily labile *cis*-regulatory interactions may evolve alongside diversification of condition-specific traits, potentially modifying the activity of conserved developmental pathways over short evolutionary timescales.

## Author summary

In this work, we examine a central question in evolutionary biology: how environmental and genetic variation is converted into morphological differences during development. In our study system, *Onthophagus* horned dung beetles, horn shape and size vary tremendously across species, sexes, and nutritional conditions, making them ideal for studying condition-responsive trait development. We focus on comparing the characteristics of regulatory elements in horn tissue, such as enhancers and promoters, to understand better how gene expression changes during developmental evolution. We show that mutations have accumulated within potentially important developmental regulatory elements across species with divergent horn shapes and nutritional responsiveness. We find that hundreds of elements differ in chromatin accessibility between conditions, including many elements that occur in only a single species. Finally, we find that many candidate regulatory elements harbor binding motifs for key transcription factors, including those involved in bone morphogenetic protein signaling, and we validate that members of this pathway play a complex role in horn development and, potentially, diversification. More broadly, our findings suggest that the evolution of condition-sensitive traits may proceed through relatively rapid changes to regulatory element properties, enabling conserved developmental pathways to generate novel morphologies.

## 1. Introduction

Gene regulatory networks (GRNs) describe the molecular interactions within a cell that control gene expression, thereby determining a cell’s function and identity. Accordingly, GRNs play critical roles in morphogenesis, tissue differentiation, and developmental evolution [1]. Research characterizing the molecular basis for morphological innovations has revealed that highly conserved gene networks are frequently co-opted across body regions and developmental stages to establish new traits [2,3]. For instance, gene networks described initially for their roles in embryonic and appendage patterning are also implicated in the origination of butterfly wing spots [4,5], beetle horns [6], and fruit fly phallic projections [7]. However, the initiation of a novel trait represents just the beginning of morphological diversification, often occurring deep in a lineage’s evolutionary history (tens to hundreds of millions of years ago). Less is known about how gene regulatory networks (GRNs) evolve over short evolutionary timescales to drive subsequent morphological innovation and differentiation after a trait has been established. Such knowledge is especially scarce for sexual dimorphisms and environment-sensitive development — contexts that encompass many of the most rapidly evolving traits in the animal kingdom [8,9].

Over the past two decades, *Onthophagus* dung beetles have emerged as a robust system for addressing these and related questions. This hyper-diverse genus of more than 2000 extant species [10] is perhaps best known for the extraordinary diversity of head horns among its members. These secondary sexual weapons display remarkable variation in size, shape, number, and position both across and within species (Fig 1a), highlighting the dorsal insect head as a hotspot for morphological innovation [11,12]. Furthermore, horn exaggerations, gains, and losses have occurred repeatedly in disparate lineages [13], suggesting that intense selective pressures may be driving the rapid and ongoing diversification of this novel trait. The transcriptomic basis for horn variation has been analyzed in depth in *Onthophagus* [12,14–17], and collectively, these studies identified many key genes whose expression and developmental function vary across and within species.

**Fig 1:**
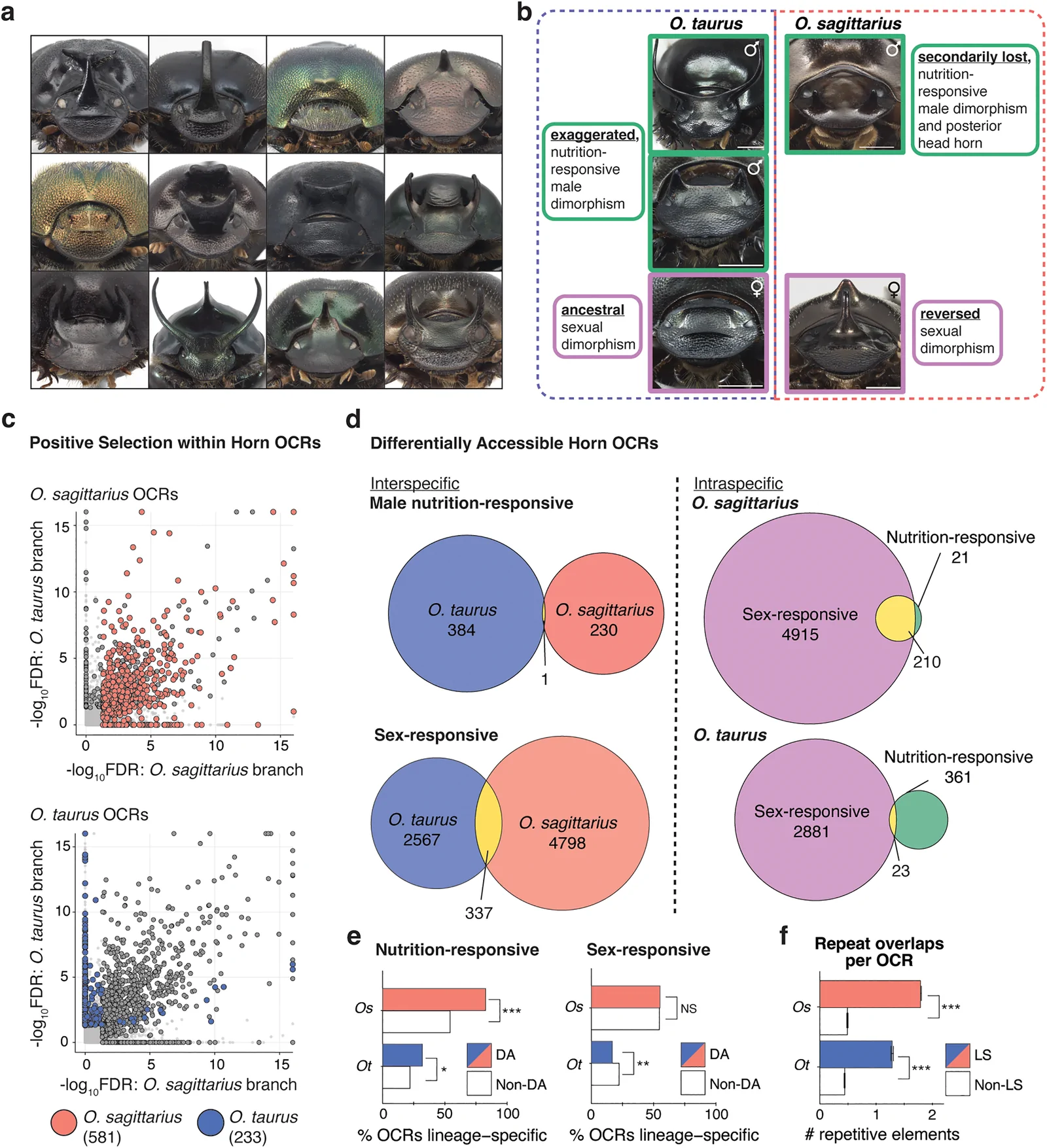
Extraordinary diversity in onthophagine horn morphology correlates with distinct modes of *cis*-regulatory evolution. (a) Portraits of *Onthophagus* dung beetle species exhibiting an array of horn morphologies. (Left to right): top: *O. ferox*, *O. sp.* (undescribed- Vietnam), *O. aciculatus*, *O. fracticornis*; middle: *O. auriceps*, *O. lunatus*, *O. binodis*, *O. australis*; bottom: *O. quadridentatus*, *O. mowhoti*, *O. trituber*, *Digitonthophagus gazella* (outgroup). (b) Male and female individuals of the focal species of this study, *O. taurus* and *O. sagittarius*, highlight the derived traits of each species. (c) Evidence for positive selection within open chromatin regions (OCRs) identified in pupal head horn tissue of *O. sagittarius* (top) and *O. taurus* (bottom). Adjusted p-values attained from the likelihood ratio test comparing the alternative (positive selection) and null (neutral selection) model fit. Red indicates positive selection along the *O. sagittarius* branch, blue indicates positive selection along the *O. taurus* branch. For clarity, we have colored only OCRs with evidence of species-specific selection from that species’ dataset; see S4 Data for the complete annotation of results. (d) Number of differentially accessible (DA) pupal head horn OCRs. *Left:* Differentially accessible OCRs between species according to nutritional state (top) or sex (bottom) in *O. sagittarius* (red), *O. taurus* (blue), or both species (yellow). *Right*: Differentially accessible OCRs within species according to sex (purple), nutritional state (green), or both (yellow) in *O. sagittarius* (top) or *O. taurus* (bottom). (e) Proportion of lineage-specific pupal horn OCRs that are differentially accessible between male nutritional states (left) and sexes (right) in *O. sagittarius* (*Os*) and *O. taurus* (*Ot*). (f) Number of repetitive element overlaps per OCR at lineage-specific and shared loci in *Os* and *Ot*. Pearson’s chi-squared test: p-value < 1e-5 (*); p-value < 1e-10 (**); p-value < 1e-15 (***). Scale bar: 1mm.

While horn expression differences between and within horned beetles have been examined in depth, the mechanisms by which associated genetic architectures (i.e., *cis*-regulatory elements) facilitate such expression changes and subsequent morphological evolution over short evolutionary timescales remain comparatively understudied, in both *Onthophagus* and other developmentally plastic taxa more broadly [18]. Historically, *cis*-regulatory elements (CREs) have been predicted to be subject to fewer pleiotropic constraints than coding sequences, as their activity is often restricted to specific cell types and developmental stages [19–21]. However, recent empirical work testing the relative contribution of *cis*- and *trans*-acting factors to phenotypic evolution has challenged this perspective [22]. For example, a single enhancer may operate in multiple tissues or developmental stages [23,24], affect the expression of multiple genes [25], or even modify genomic architecture via changes to chromatin looping [26]. Thus, comparing *cis*-regulatory characteristics in natural systems that exhibit variation in plastic trait development has the potential to provide new insights into the relative contributions of these elements to changes in developmental regulation, especially considering that plastic traits are often restricted to specific tissue types, developmental periods, or life history stages.

In this study, we investigate the genetic basis for horn variation in *Onthophagus* dung beetles using a series of comparative genomic and developmental assays. Specifically, we aim to identify and connect differences in key properties of open chromatin regions (accessibility, sequence, and gain/loss) to the evolution of exaggerated nutrition-responsive horn development in *Onthophagus taurus* as well as the secondary loss of male nutrition responsiveness in the closely related (~4 million years diverged) *O. sagittarius*, a species which simultaneously evolved striking sex-*reversed* horn development (Fig 1a). We characterize a dynamic regulatory landscape that covaries with environmental and genetic contexts, does so divergently across two taxa, and examine how a conserved signaling network may be contributing to within- and among species diversity in the development of secondary sexual weaponry.

## 2. Results and discussion

### 2.1. Recent evolution of the horn regulatory landscape correlates with intraspecific variation

We predicted that CREs may play an especially important role in mediating the variation in head horn development found within and among dung beetle species given the variability and rate at which this trait evolves among closely related species. Using ATAC-sequencing, we identified over 160,000 open chromatin regions (OCRs) in primordial, epithelial head horn tissue, which then allowed us to compare rates of sequence evolution and chromatin accessibility between *O. taurus* and *O. sagittarius* (S1 Data). By comparing homologous tissues from these two closely related species, we could begin to characterize the regulatory dynamics underlying the diversification of this trait.

First, we permuted substitution rate estimates for homologous OCRs and neutrally evolving sequence proxies to test for evidence of branch-specific positive selection in either species [27,28]. We detected such evidence in 581 OCRs in *O. sagittarius* and 233 OCRs in *O. taurus* (Fig 1c: top and bottom, respectively)*,* composing 1.39% and 0.50% of the homologous OCRs tested in each species. This roughly 2.8-fold higher proportion of sites under positive selection in *O. sagittarius* is conspicuous and may be related to unique evolutionary changes in horn formation observed in this species. Specifically, this species has both 1) lost male horn plasticity *and* 2) acquired sex-reversed horn development (females grow posterior head horns, males do not; see Fig 1b). Alternatively, evidence of selection at many more OCRs along the *O. sagittarius* branch relative to *O. taurus* could instead reflect constraint on these sites in *O. taurus* that is absent in *O. sagittarius* (i.e., CREs especially important for maintaining male nutritional plasticity or ancestral sex-based differences). Nonetheless, because many OCRs are predicted to overlap putative CREs, branch-specific mutation accumulation suggests evolutionary changes in gene regulation within this cell type, potentially through altered transcription factor binding to these elements (see Section 2.2).

Next, we compared chromatin accessibility of OCRs from head horn epithelial cells across two axes of intraspecific variation, nutrition-responsive male plasticity (high vs. low nutrition) and sexual dimorphism (males vs. females), to determine how trait variation within a species correlates with chromatin configuration (Fig 1d). In *O. taurus* only, high and low nutrition produces discrete morphs called “majors” and “minors”. Of note, the number of OCRs analyzed here is greater than in the previous positive selection tests because accessibility analyses are not restricted to homologous OCRs.

When comparing high and low nutrition males, we identified 385 (0.54% of 71,820) differentially accessible OCRs in the highly nutrition-responsive *O. taurus*, and 231 (0.25% of 94,271) in the nutritionally much less responsive *O. sagittarius* (Fig 1d, left, top). Conversely, when analyzing sex-responsive OCRs, we found 2,904 (4.04%) differentially accessible OCRs between males and females in *O. taurus*, and 5,135 (5.45%) in *O. sagittarius* (Fig 1d, left, bottom). These proportional differences mirror each species’ recent exaggeration of context-dependent development: *O. taurus* males exhibit both a pronounced nutrition-dependent polyphenism and possess more nutrition-responsive OCRs, whereas in *O. sagittarius*, females, rather than males, develop striking posterior head horns and possess more sex-responsive OCRs (Fig 1b). While the correlation between differential accessibility and exaggerated horn phenotypes in either species raises the possibility that chromatin configuration may play a pivotal role in regulating intraspecific variation of this trait, many explanations for these accessibility differences beyond regulation of horn development exist. This is especially pertinent given that chromatin accessibility alone is a permissive rather than instructive feature of transcription factor binding and, by extension, gene regulation [29]. For example, chromatin accessibility differences in horn epithelial cells may have arisen in progenitor cells during an earlier developmental process unrelated to horn morphogenesis or reflect drift between species. Further comparisons across more species and functional studies assessing putative CRE activity will help clarify the relationship between chromatin configuration and changes in horn development.

Interestingly, only a single (1) homologous OCR was identified as nutrition-responsive among high and low nutrition males of *both* species (Fig 1d, yellow). In contrast, 337 homologous OCRs are sex-responsive in both species. We propose that this shared set of sex-responsive OCRs may reflect an ancestral set of regulatory interactions governing horn sexual dimorphism in dung beetles. Further evidence for a distinct nutritional regulatory architecture in *O. taurus* emerges from a comparison of the overlap between nutrition- and sex-responsive OCRs (Fig 1d, right). In *O. taurus*, nutrition- and sex-responsive OCRs are almost entirely distinct, whereas in *O. sagittarius*—a species lacking a nutrition-responsive horn phenotype—they overlap almost completely. This suggests that developmental horn OCRs responsive to nutritional plasticity (or its absence) are derived and may underpin species-specific variation in nutritional plasticity.

Lastly, we examined OCR conservation (presence or absence of putative regulatory regions) across ten beetle species to assess how the lineage specificity of OCRs covaries with differential chromatin accessibility. At a genome-wide scale, we found an abundance of differentially accessible, lineage-specific OCRs, especially in nutrition-responsive (*O. taurus* and *O. sagittarius*) and sex-responsive regions (*O. sagittarius*, only) (Fig 1e). Furthermore, we detected far more lineage-specific sites in *O. sagittarius* head horn regions compared to *O. taurus,* regardless of differential accessibility (Fig 1e, white bars: ~ 54% vs ~ 23%). This increase in lineage-specific OCRs in *O. sagittarius* may be related to its recent genome expansion and a burst of transposable element (TE) activity, primarily due to DNA transposons (S1 Fig). This is further supported by a significantly higher abundance of TEs within lineage-specific OCRs compared to OCRs present in both species (Fig 1f). Since TEs are widely thought to generate evolutionary changes in gene regulatory interactions [30–32], it is plausible that TE activity has played a key role in generating new regulatory elements in dung beetle genomes as well. Still, functional validation of the activity of these lineage-specific elements will be necessary to determine what role, if any, they play in horn development and diversification.

### 2.2. Context-dependent development covaries with differential transcription factor motif enrichment

We found that many OCRs showing evidence of species-specific positive selection or intraspecific differential accessibility were located near key developmental regulatory genes (e.g., *doublesex*, *limb expression 1*, *paired box 6*, *sox 5*, *osa*) (Fig 2a). To test if differential accessibility or evidence of positive selection within OCRs correlated with differential expression of nearby genes at a genome-wide scale, we cross-listed this dataset with two previously published RNA-seq datasets in *O. taurus* that compared sex [12] and nutrition responses [17] in the same tissue. While we did not detect significant genome-wide association of differential accessibility (sex: p > 0.08; nutrition: p > 0.88) or positive selection (sex: p > 0.26; nutrition: p > 0.76) with differential expression (Fisher’s exact test, two-sided), we did identify several new candidate (*msx*, *lix1*, *pou6f2*) and known (*dsx*) horn developmental transcription factors with differential expression and nearby OCRs with differential accessibility or evidence of positive selection (see S2 Data).

**Fig 2:**
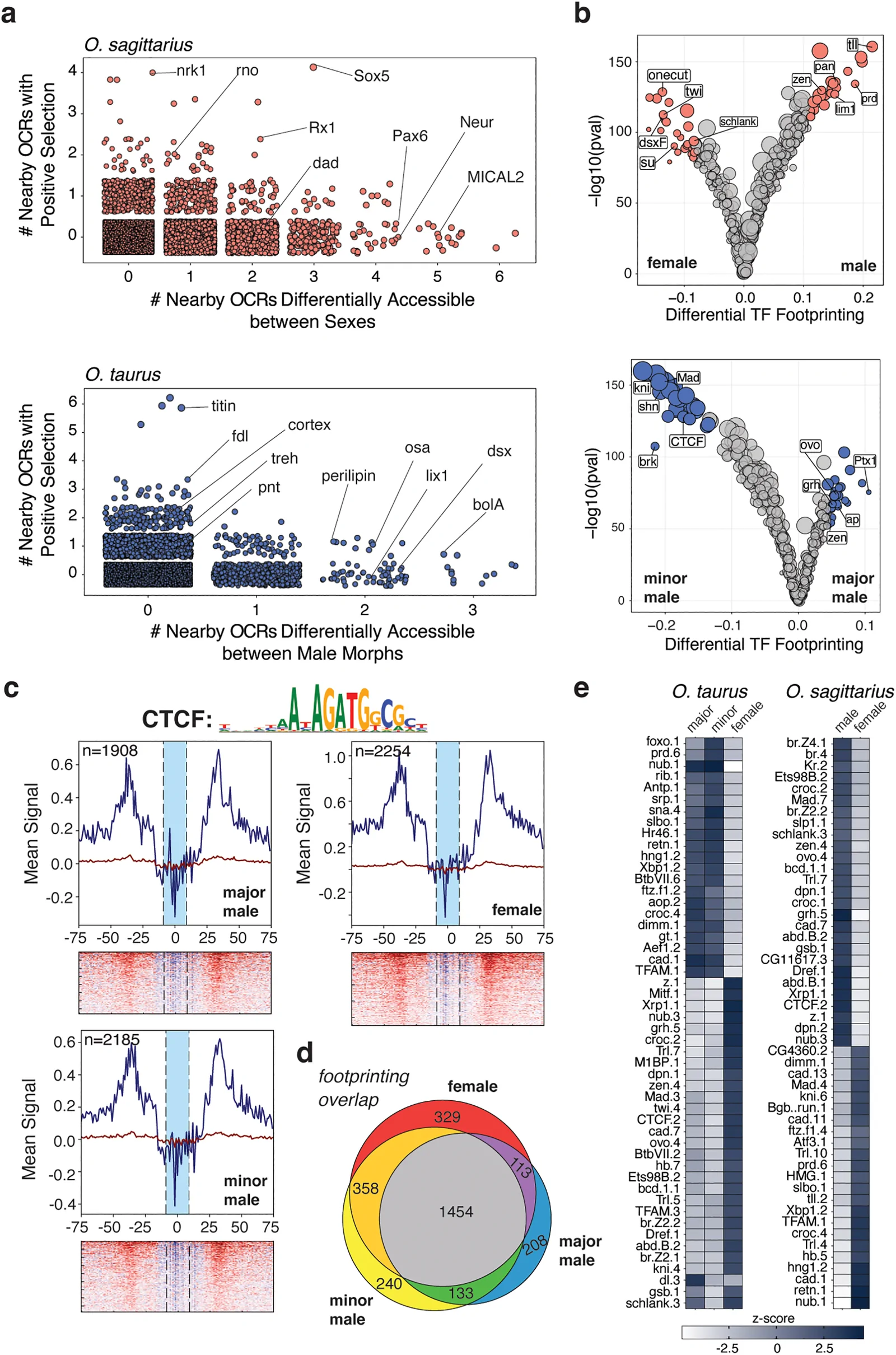
Alternative suites of transcription factor motifs covary with inter- and intraspecific variation in the horn regulatory landscape. (a) Number of OCRs with intraspecific differential accessibility and branch-specific positive selection in *O. sagittarius* (top) and *O. taurus* (bottom) within 20kb of gene translation start sites. (b) Differential predicted transcription factor footprinting in pupal head horn tissue between sexes in *O. sagittarius* (top) and nutritional state in *O. taurus* (bottom) using TOBIAS (Bentsen et al., 2020). Circle size scales to the relative number of TF footprints. (c) Transcription factor footprinting of CCCTC-binding factor (CTCF) binding in *O. taurus* major males, minor males, and females. (d) Intraspecific variation in shared and unique predicted CTCF footprints within pupal head horns of *O. taurus*. e) Intraspecific differences in transcription factor binding motif profiles within pupal horn OCRs of *O. sagittarius* and *O. taurus* using GimmeMotifs (Bruse & Heeringen, 2018). Z-score represents the ranking of a TF’s binding signal within a sex or nutritional state relative to the mean signal of all TFs in the dataset.

Next, we identified and screened 407 transcription factor (TF) binding motifs within these OCRs (enrichment analysis) and tested how differential TF footprinting (a computational prediction of TF binding activity) correlates with variation in horn morphology. From the TF motif enrichment analyses, we found that binding motif profiles within horn OCRs were often distinct between sexes (*O. sagittarius*, *O. taurus*), developmental nutrition (males of *O. taurus*), and species (Fig 2e). For instance, the most enriched TF motif in minor males compared to major males or females in *O. taurus* belongs to *foxo*, a key regulator of insulin signaling previously implicated in mediating horn-body size allometry in this species [33]. When comparing significantly enriched TF motifs across species, sex, and nutrition, we found that the hornless condition, regardless of sex or species, shared the most enriched TF motifs of any individual or multi-condition comparison (14 TFs: *O. sagittarius* males, *O. taurus* females) (S2 Fig), suggesting a conserved regulatory repertoire may exist to repress horn formation across species and developmental contexts. Conditions with horn development (*O. taurus* major males, *O. taurus* minor males, and *O. sagittarius* females), on the other hand, shared 6 significantly enriched TF binding motifs within developmental horn OCRs (S4 Data, S2 Fig).

We then integrated motif data with chromatin accessibility at single-base resolution to detect TF footprinting signatures across sexes or developmental nutrition. In this approach, a reduced accessibility signal over a TF binding motif within a broader region of high accessibility enables prediction of potential TF binding activity that is unattainable by sequence analysis alone [34]. For example, CTCF is a highly conserved zinc finger transcription factor and a key regulator of chromatin structure and gene expression in metazoans [35]. We observed strong CTCF footprinting signatures in developing head horn epithelial cells, with differences in both the number and location of footprints between sexes and nutritional states (Fig 2c, 2d). Notably, we detected 358 CTCF footprints unique to *O. taurus* minor males and females, compared to 133 shared sites between major and minor males (Fig 2d). This result further suggests that chromatin configuration relates to the evolution of condition-dependent gene regulation. We also observed intraspecific variation in TF footprinting scores within horn OCRs associated with each species’ derived trait (male nutrition in *O. taurus*, sex-based reversal in *O. sagittarius*) (Fig 2b). Because these TF footprinting profiles vary considerably between sexes and developmental nutrition, it is plausible that modifying TF-DNA regulatory interactions was an important step during the evolution of these condition-responsive phenotypes.

While our ATAC-seq analyses detected significant TF motif enrichment of numerous developmental TFs (e.g., *twist, foxo*) and pathways (e.g., Wnt signaling, axis polarity genes) within putative horn CREs, the most notable finding was the abundance of binding site motifs for proteins associated with the bone morphogenetic protein (BMP) signaling pathway (Fig 2b, 2e). For example, transcription factor footprinting analysis detected significantly more *zerknüllt* (*zen*) binding events in head horn OCRs of *O. sagittarius* males compared to females, as well as in *O. taurus* major males compared to minor males (Fig 2b). In addition, *mothers against dpp* (*mad*), *schnurri* (*shn*)*,* and *brinker* (*brk*) each exhibited greater TF footprinting signatures in minor compared to major males in *O. taurus* (Fig 2b: bottom). High condition-specific motif enrichment of *mad* and *zen* was also observed at a genome-wide scale in both species (Fig 2e). The prevalence of BMP-associated TF motifs within head horn OCRs prompted us to investigate this pathway in developing horn tissue in greater detail.

### 2.3. BMP-associated proteins are co-opted into recent inter- and intraspecific diversification processes of beetle horns

Proteins in the BMP pathway, part of the transforming growth factor-β (TGF-β) superfamily, form an ancient signaling cascade fundamental to embryonic and appendage development, dorsal-ventral patterning, tissue specification, and various morphogenetic processes across metazoans [36] (Fig 3a). To further understand this pathway’s role in beetle horn diversity, we explicitly searched horn OCRs showing evidence of species-specific positive selection or differential accessibility between sexes or nutritional states for binding motif enrichment of seven TFs involved in BMP signaling: *mad, medea* (*med*)*, brk, optomotor-blind* (*omb*)*, pannier* (*pnr*)*, shn,* and *zen* (Fig 3b).

**Fig 3:**
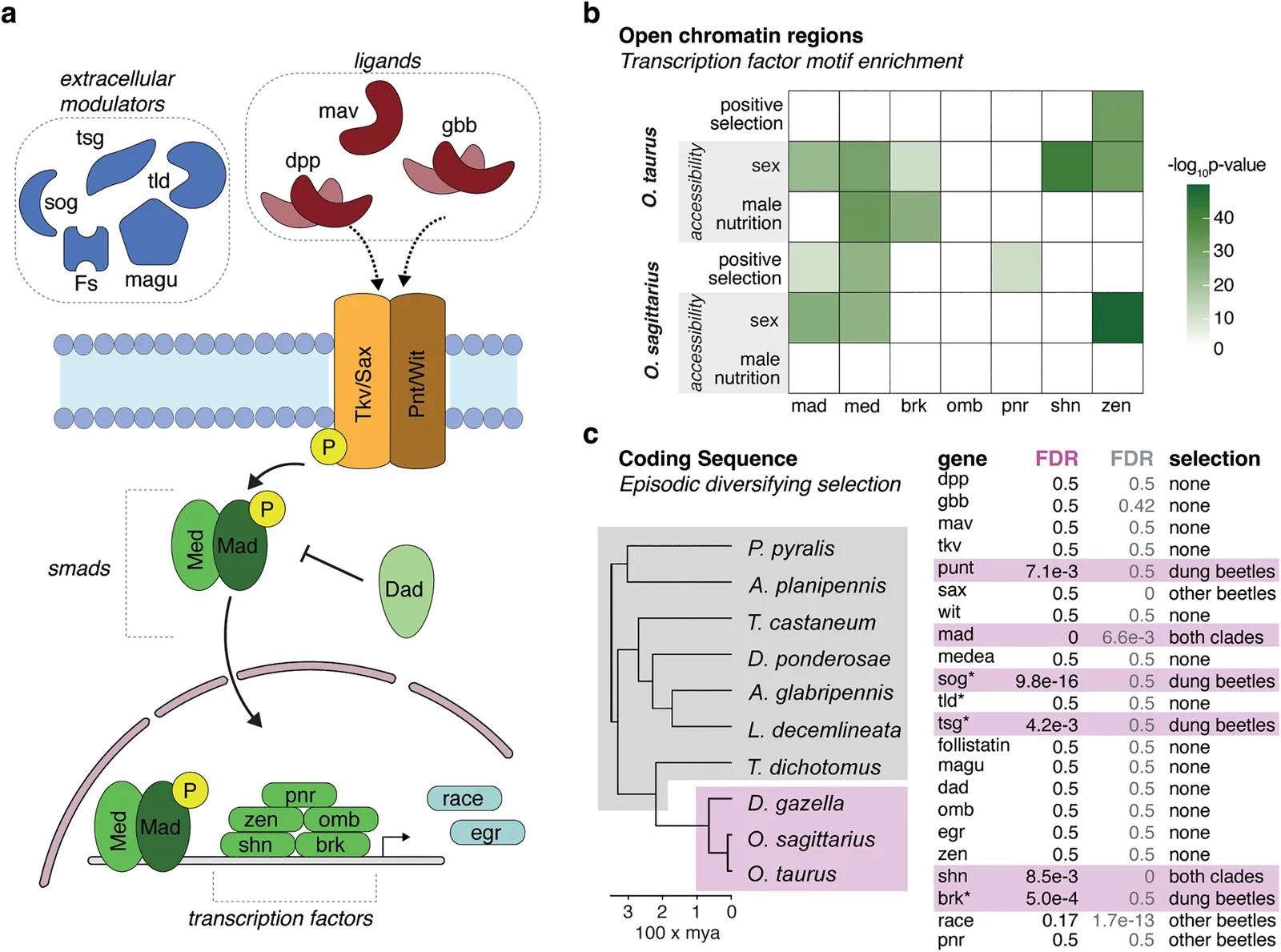
Pervasive signatures of positive selection detected within the bone morphogenetic protein pathway in horned dung beetles. (a) Model of bone morphogenetic protein (BMP) signaling. (b) BMP transcription factor binding motif enrichment within OCRs that are differentially accessible between sexes, nutritional states, and exhibit evidence of lineage-specific positive selection in *O. taurus* and *O. sagittarius*. (**c)** Evidence of episodic diversifying selection within the coding sequence of BMP genes in three dung beetle species (purple) relative to seven other beetle species (gray).

We found significant enrichment for each TF binding motif in at least one OCR set compared to other horn OCRs, except *omb*. Conversely, male nutrition-responsive horn OCRs in *O. sagittarius* showed no enrichment for BMP-associated TFs, consistent with this species’ independent loss of horn plasticity. Of note, whereas *mad* and *med* are BMP-specific signal transducers, the other five TFs examined here operate across multiple regulatory networks. Therefore, our analysis does not rule out the involvement of other signaling pathways; in fact, both the Wnt and hedgehog pathways have been shown to play central roles in beetle horn morphogenesis [6,37]. Still, the prevalence of BMP-associated TF binding motifs in OCRs exhibiting positive selection or condition-dependent accessibility, alongside evidence of diversifying selection in the coding sequences of BMP extracellular modulators and receptors (below), raised the possibility that this pathway may be critical in modulating inter- and intraspecific regulation of this trait.

We scanned the coding sequences of these seven TFs and twelve other proteins involved in BMP signaling for evidence of episodic diversifying selection in dung beetles (Fig 3a, 3c). To do so, we compared non-synonymous to synonymous mutation rates [38] within coding sequence alignments from three horned dung beetle species (*O. taurus*, *O. sagittarius*, and the more distantly related *D. gazella*) as well as seven additional beetle species (alignments from ref [39].) (Fig 3c). We detected evidence of diversifying selection in six BMP-associated signaling genes in dung beetles (*punt*, *mad*, *shortened gastrulation* (*sog*), *twisted gastrulation* (*tsg*), *shn,* and *brk*), though *mad* and *shn* showed evidence of diversifying selection in non-dung beetles as well. These findings indicate that developmental CREs are not only enriched for BMP-associated TF binding motifs (Fig 3b) but also that non-synonymous mutations have accumulated in the coding sequences of several BMP-associated genes. Additionally, reanalysis of previously published RNA-seq data comparing head horn tissue expression between *O. taurus* major and minor males [17] identified five differentially expressed BMP-associated pathway genes (of 23 tested: 21.7%; see S1 Table), which is proportionally greater than all differentially expressed genes from this study (815/7,640: 10.7%). Taken together, this suggests a recent and potentially adaptive deployment of the BMP signaling pathway within this clade during horn evolution.

To test this prediction, we investigated the functional roles of BMP-associated signaling components in beetle horn morphogenesis and diversification by conducting a series of expression knockdown experiments targeting three critical BMP pathway members *— tsg*, *sog*, and *brk —* in *O. taurus* and *O. sagittarius*. *Tsg* and *sog* were tested first because both genes encode extracellular modulators of the BMP signaling cascade. Thus, we predicted that knockdown of these genes’ expression would be highly consequential and more readily detectable. Both genes also showed evidence of episodic diversifying selection in dung beetles. *Brk* was targeted due to our discovery of an abundance of *brk* binding site motifs within differentially accessible head horn OCRs (Figs 2b; 3e) and similar evidence of episodic diversifying selection in its coding sequence. We performed RNA-interference (RNAi) on male and female third instar larvae, the developmental stage immediately preceding horn shape specification [40], and assessed adult horn length and shape after eclosion (Fig 4).

**Fig 4:**
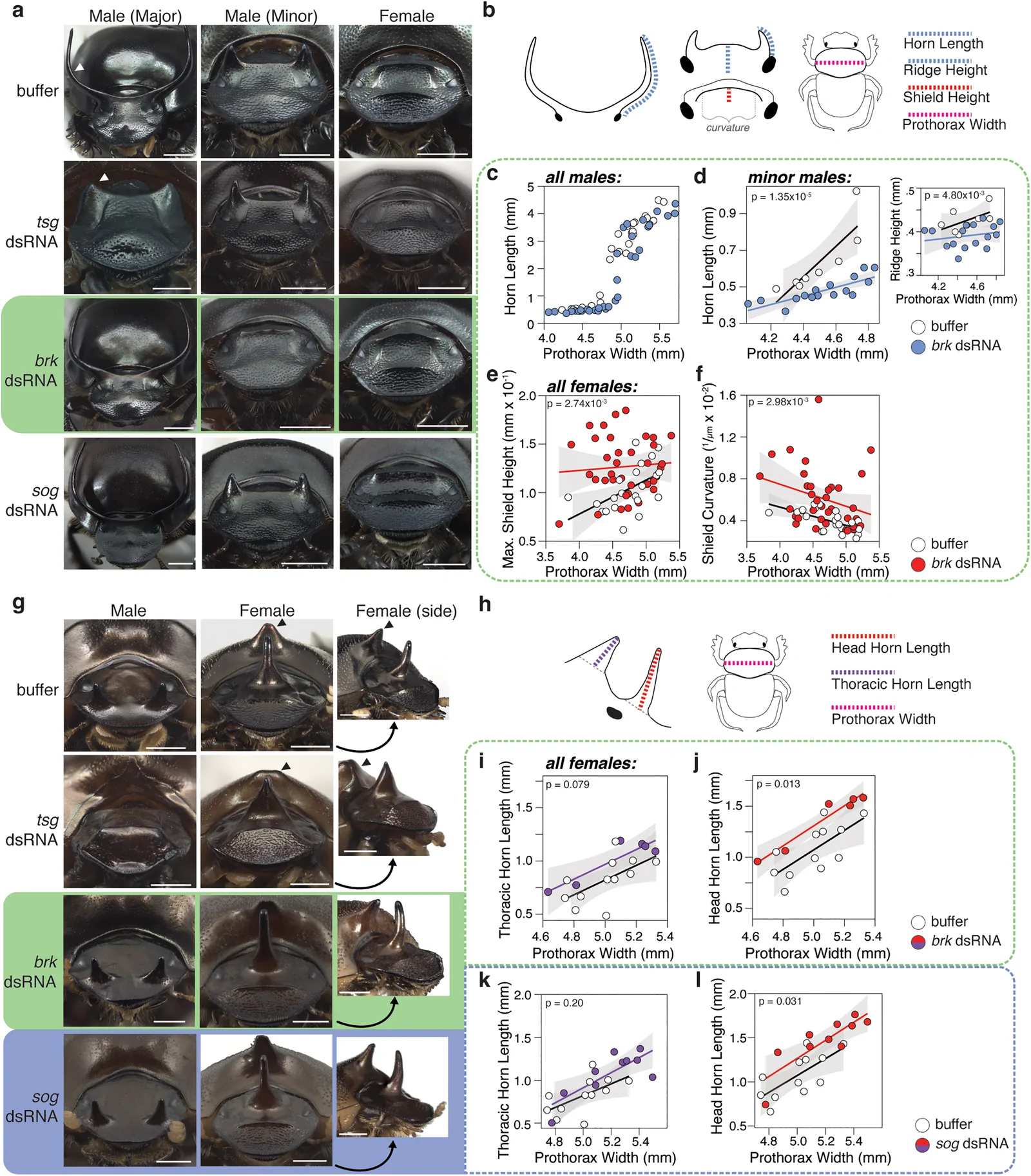
The BMP signaling pathway has been co-opted into discrete developmental conditional and spatial developmental networks to facilitate rapid horn diversification. Expression knockdown results of *twisted gastrulation* (*tsg*), *brinker* (*brk*), and *shortened gastrulation* (*sog*) in *O. taurus* (a) and *O. sagittarius* (g) via RNA-interference. Illustration of morphometric measurements in *O. taurus* (b) and *O. sagittarius* (h). Horn and prothorax measurements of all males (c) and minor males (d) in *O. taurus brk* RNAi experiments. Inset of (d): minor male prothorax and ridge height measurements. Measurements of prothorax width, maximum ridge height (e), and ridge curvature (f) in *O. taurus* female *brk* experiments. Measurements of prothorax width, thoracic horn (i), and head horn (j) length in *O. sagittarius brk* RNAi experiments. Measurements of prothorax width, thoracic horn (k), and head horn (l) length in *O. sagittarius sog* RNAi experiments. Empty circles denote control (buffer) injected individuals, colored circles denote double-stranded RNA injected individuals for all experiments. P-values are derived from analysis of covariance (ANCOVA) tests modeling body size, horn length, and dsRNA injection. Scale bar: 1mm.

In *O. taurus*, expression knockdown of the BMP protagonist *tsg* did not result in any detectable posterior head phenotype in minor males or females. However, major males exhibited a pronounced decrease in horn size, with broad, rudimentary horns compared to size-matched controls. This phenotype is reminiscent of the short-horned phenotype normally seen in low-nutrition males (Fig 4a). In contrast, knockdown of the BMP repressor *brk* did not affect horn length in major males (S3 Fig) or overall horn-body size allometry (Fig 4a–4c). Instead, minor males injected with *brk* dsRNA showed a modest but significant and consistent reduction in both horn length and ridge height across all body sizes (Fig 4a, 4d). Interestingly, the posterior heads of *O. taurus* females exhibited an opposing developmental response to *brk* expression knockdown, with increased posterior ridge height and curvature due to excess growth in the medial region of this structure (Fig 4a, 4e, 4f). Finally, knockdown of another extracellular modulator, *sog*, did not produce any measurable differences in posterior head morphology in either sex (Fig 4a; bottom). Taken together, these results provide evidence that BMP-associated genes in *O. taurus* may function in discrete developmental contexts (major male, minor male, females) to impact the structure of a single trait.

Given the evidence that BMP-associated genes regulate horn morphology in *O. taurus*, we replicated these experiments in *O. sagittarius* to investigate potential evolutionary differences in this pathway during horn development (Fig 4g, 4h). Knockdown of *tsg* expression in *O. sagittarius* females resulted in a pyramidal-shaped posterior head horn, in contrast to the thin, cylindrical horns of controls (Figs 4g; S4). Additionally, reduction of *tsg* expression eliminated the female thoracic horn (Fig 4g, black arrow) and, in males, fused the two anterior horns into a ridge-like structure, whereas the posterior area was unaffected. Collectively, *tsg*RNAi phenotypes observed in the dorsal head of adult *O. sagittarius* tended to resemble wildtype phenotypes normally observed in the *pupal* stage (S4 Fig). During this stage, horn epithelial tissue is normally sculpted into its final adult shape through cell orientation changes and apoptosis before crosslinking and hardening of the overlying adult cuticle [41,42]. Thus, a possible mechanistic explanation for these results is that in *O. sagittarius, tsg* is required for proper epithelial sculpting of the developing pupa into the final adult structure. Our comparison of *tsg* RNAi phenotypes across the two species highlights distinct developmental periods during which BMP signaling may regulate horn morphology. Specifically, our results suggest that BMP signaling: (1) regulates prepupal growth and proliferation of horn primordia, leading to changes in pupal horn structures (e.g., reduced horns in *O. taurus* major males), as well as (2) directs the subsequent remodeling of pupal epithelial tissues to establish final adult horn size and shape (e.g., narrow versus broad horns in *O. sagittarius* females).

In partial contrast, knockdown of *brk* expression in *O. sagittarius* females produced slightly longer head horns (Fig 4g, 4j), which parallels increased posterior medial growth observed in *O. taurus* female ridges following the same treatment (Fig 4e). We detected a similar increase in head horn length in *O. sagittarius* females following *sog* dsRNA injections (Fig 4g, 4l), albeit sample sizes are modest in both experiments. However, knocking down *brk* and *sog* expression did not significantly affect thoracic horn length in females (Fig 4g: black arrow, Fig 4i, 4k) or the size of anterior head horns in males (Figs 4g, S5), neither of which is homologous to posterior head horns (Hu et al., 2019). Taken together, these results suggest that a highly conserved developmental regulatory network has been co-opted into the development of beetle horns and may play an important role in regulating condition-dependent morphological variation across species.

## 3. Conclusions

Condition-responsive trait development generates much of the phenotypic variation found in natural populations, ranks among the fastest-evolving trait categories, and supplies important raw material for natural selection to act upon. Yet the origins and subsequent diversification of conditional trait formation within the boundaries of existing regulatory architectures remain largely unresolved. In this study, we take a genomic approach to compare the regulatory landscapes of Onthophagine dung beetle species that exhibit highly divergent forms of condition-responsive horn development (Fig 1b). We identify three classes of *cis*-regulatory differences between two species—sequence change within OCRs, shifts in chromatin accessibility, and the presence of lineage-specific OCRs—each of which correlates with the recent emergence of species-specific conditional trait formation (Figs 1c–1e; 2). However, this study’s insight into CRE evolution is limited by the comparison of only two species and genomic-level associations, precluding further causal inferences about CRE activity and phenotype. Future work aimed at functionally validating CRE function and/or including additional species is necessary to establish the extent to which these elements drive phenotypic evolution.

By integrating transcription factor footprinting and motif analyses (Fig 2d–2e), we predicted potential modifications to developmental pathway activity, including the BMP signaling pathway, which may be linked to the regulation and rapid evolution of beetle head horns. Evidence of positive selection was found in both the coding regions of BMP pathway genes and the noncoding regions containing BMP-associated protein binding motifs, suggesting this pathway may play a significant role in both inter- and intraspecific variation of this trait (Figs 2, 3). Previous RNA-sequencing of the same tissue also identified BMP members as important horn regulatory factors, but their significance was overlooked among a list of over 800 candidate genes. The comparative *cis*-regulatory analyses we present here offer greater resolution into associated regulatory dynamics (chromatin accessibility, mutation, putative CRE gains and losses, TF motif analysis) and set the stage for a more nuanced identification of candidate genes than coding sequence analyses alone would allow. The value and power of such a multipronged approach to investigate the developmental genetic basis of phenotypic innovation and diversification has recently been demonstrated in the study of sexual wing dimorphisms in cockroaches and other hemimetabolous insects [43].

RNAi-mediated transcript knockdowns confirmed that BMP signaling likely has essential and distinct functions in developing sex- and nutrition-specific horn phenotypes (Fig 4). However, due to the pleiotropic nature of BMP signaling and the various phenotypes observed in these experiments, a more comprehensive assessment of BMP function in these species is needed to better characterize the mechanisms by which this pathway is integrated into horn development. For example, in *O. taurus*, reducing *brk* expression produced minor males with smaller horns and females with taller ridges, while in *O. sagittarius*, it increased horn length in females. Lateral-medial variation in *brk* expression may explain these results — medial structures (female ridge, horn) showed enhanced growth, while lateral structures (male horns) showed reduced growth. Alternatively, *brk* may exert opposing effects on growth in males compared to females. Future research aimed at characterizing the binding targets (e.g., CHiP-seq, CUT&RUN) and spatial expression domains of this gene and other BMP members will deepen our understanding of this pathway’s role in horn developmental evolution. More broadly, our findings provide an additional example highlighting the modularity of the BMP pathway in mediating morphological diversification among closely related species.

## 4. Materials and methods

### 4.1. Animal husbandry and rearing

Adult *Onthophagus taurus* beetles were collected from Chapel Hill, North Carolina, USA (35.936, -79.128), and adult *O. sagittarius* beetles were collected at Kilcoy, Queensland, Australia (-26.951, 152.605). Beetles were kept in captivity in a sand-soil mixture as described in ref [44]., including a 16:8 light-dark cycle, ambient temperature at 24ºC for *O. taurus* and 28ºC for *O. sagittarius*, and provided a diet of homogenized cow manure. Six females and three males were bred over a week, and first instar larvae were transferred to artificial brood balls in 12-well plates (for the protocol, see ref [45].). Depending on nutritional treatment, plates were loaded with manure from either grass-fed cows (high nutrition) or hay-fed cows (low nutrition) and reared at either 24ºC (*O. taurus*) or 28ºC (*O. sagittarius*).

### 4.2. ATAC-sequencing sampling and library preparation

We aimed to compare chromatin accessibility in the pupal dorsal posterior head epithelia of males and females developing under high and low nutritional conditions in both species. For *O. taurus,* these data were retrieved from ref. 39 and include four high-nutrition male samples, five low-nutrition male samples, and seven female samples. To generate a comparable dataset in *O. sagittarius*, first instar larvae were reared through the late third instar stage in temperature-controlled incubators. Then, plates were checked every 12 hours for pupating individuals. Upon pupation, live dorsal head epithelial tissue was dissected in sterile 1X PBS (phosphate-buffered saline) in 3-well glass dissection plates as described in Casasa et al., 2020. Immediately after dissection, approximately 50,000 cells (determined via counting DAPI-stained cells on a hemocytometer) were transferred to a 1.5 mL conical tube for ATAC-sequencing [46] using the Omni-seq protocol [47].

We collected five biological replicates for each combination of sex (male vs. female) and nutrition (high vs. low), resulting in a total of 20 samples. Samples whose sequencing library required more than 20 PCR cycles to amplify were discarded (four samples), as this indicates poor library construction and/or complexity; a final set of 16 samples remained, including four high nutrition males, five low nutrition males, three high nutrition females, and four low nutrition female samples. Samples were sequenced on an Illumina NextSeq 550 instrument to acquire 50 bp paired-end reads (same as *O. taurus*).

### 4.3. ATAC-sequencing data analysis

ATAC-seq reads from both species were processed using identical parameters. Reads were trimmed using Trimmomatic v. 0.39 (parameters: leading: 10, trailing: 10, slidingwindow: 4:15, minlen: 25) [48] and aligned to their respective genome assemblies using bowtie v.2.2.5 [49]. Properly paired alignments were filtered for a minimum MAPQ score of 20, and PCR duplicates were removed using Picard Tools v. 2.18.7 (broadinstitute.github.io/picard). After all filtering steps, an average of 15.15 million and 14.2 million reads per sample remained for *O. sagittarius* and *O. taurus*, respectively. ATAC-seq peaks were identified using MACS2 (parameters: --nomodel --keep-dup = auto --shift 100 --extsize 200 -g [species’ genome size] -f BAMPE -q 0.05) [50]. For each species, peaks (hereafter open chromatin regions: OCRs) were merged into consensus OCR sets using bedtools v.2.30 [51], and accessibility counts were estimated using the bedtools *multicov* command. Accessibility counts were imported into R for statistical analysis.

After calculating accessibility counts for pupal epithelia, OCRs with very low accessibility were removed (required CPM > 3 in at least four samples), resulting in 71,820 and 94,271 OCRs in *O. taurus* and *O. sagittarius*, respectively. Differential accessibility between sexes and nutritional state was estimated using DESeq2 [52], and sites with a fold-change greater than 2 between conditions were considered significantly differentially accessible, provided they had a false-discovery rate of less than 0.1. Given the exploratory nature of this analysis, a more lenient FDR threshold of 0.1 was selected; however, overall differential accessibility patterns and our conclusions remain unchanged under a more stringent threshold of 0.05 (S3 Data). In both species, fewer than 0.05% of OCRs were differentially accessible between high and low nutrition females, so we combined these samples into a single “female” group for subsequent analyses (seven biological replicates per species). Accessibility counts (raw and filtered) are available in S1 Data. Differential accessibility analysis results are available in S3 Data.

### 4.4. Estimating evidence of positive selection in the non-coding genome

To measure branch-specific signatures of positive selection within non-coding OCRs, we followed the “*adaptiphy*” pipeline for global tests of natural selection [28]. First, whole genome alignments were generated with Progressive Cactus v.2.3 [53] between *O. taurus*, *O. sagittarius*, and *Digitonthophagus gazella* (the only other dung beetle species within the Scarabaeinae subfamily with a genome assembly). From this alignment, orthologous regions for each OCR across species were extracted and saved in FASTA format. Alignments were filtered to require at least 50 contiguously aligned base pairs between all species using the *pruning.py* and *filtering.py* scripts of *adaptiphy*. To generate a neutral reference for comparison, alignments of 10 orthologous, putatively neutrally evolving sites were concatenated into a single reference from a library of ~16,000 neutral sites generated via *adaptiphy*. A global rather than local neutral reference was implemented as a conservative measure to estimate elevated substitution rates, since use of a local neutral reference may be more likely to overestimate positive selection [28]. Substitution rates of aligned OCRs and neutrally evolving references were calculated using *phyloFit* [54], the ratio of which constitutes a site’s zeta score. P-values of likelihood ratio tests fitting a model of branch-specific positive selection were calculated using *HyPhy* [55]. For each OCR, tests of branch-specific positive selection were replicated 10 times against a uniquely assembled neutrally evolving reference to ensure zeta estimates and model fitting were reproducible. P-values were corrected for multiple testing using the “fdr” method, and sites with significant evidence of branch-specific positive selection were called as having a median zeta score greater than 1.5 and a corrected p-value less than 0.05. Results from positive selection analyses in the non-coding genome are available in S4 Data.

### 4.5. Transcription factor binding motif and footprinting analyses

Transcription factor binding motifs were predicted from pupal epithelial OCRs of each species using the *findMotifsGenome.pl* script of *homer* [56], and putative identities were assigned using the *compareMotifs.pl* script to search for similar motifs across each of the programs “insect” (primary *Drosophila*) databases. Differential motif enrichment was then measured between sexes and male nutritional state using the *maelstrom* command of *gimmeMotifs* v. 0.18 [57], whose input includes the filtered, annotated motifs described above and relative-log normalized accessibility counts across all samples. Within *maelstrom*, differential transcription factor binding activity was estimated using three independent regression models (Bayesian ridge, support vector regression, and multi-task lasso). These results were combined using the inverse normal rank transformation followed by Stouffer’s method to integrate into a final z-score (default) (Fig 2e). Lastly, transcription factor footprinting was conducted by inputting the same motif set and ATAC-seq alignments into TOBIAS v0.17.1 [34] (Fig 2b–2d). Transcription factor binding motif enrichment and footprinting results are available in S5 Data.

### 4.6. Lineage-specific OCRs

To identify orthologous or lineage-specific OCRs across beetle species, a whole-genome alignment was carried out on the soft repeat-masked genome assemblies of the same ten beetle species analyzed in Fig 3c using Cactus v.2.3 [53]. The HAL file generated from this whole-genome alignment, along with OCR coordinates, was input into HALPER [58] with the parameters: -max_frac 4 -min_len 30 -protect_dist 1 to identify orthologous OCRs of *O. taurus* or *O. sagittarius* dorsal head epithelia in the genomes of each of the other nine beetle species analyzed

### 4.7. Repetitive element analysis

Repetitive elements were identified and annotated within the *O. taurus* and *O. sagittarius* genome assemblies using the Earl Grey pipeline [59].

### 4.8. Estimating episodic diversifying selection within the BMP pathway

To estimate episodic diversifying selection, we implemented BUSTED [38]. Coding sequence alignments for 22 genes involved in BMP signaling were retrieved from ref. 39. We tested two separate evolutionary scenarios by denoting alternative sets of foreground branches in the analysis (Fig 3c): 1) evidence of selection in the clade encompassing three dung beetle species (purple shading); and 2) evidence of selection anywhere in the tree, including seven non-dung beetle species (gray shading). Significant evidence of diversifying selection was supported by an FDR less than 0.05 in one of the hypothesis tests.

### 4.9. RNA-interference and morphometrics measurements

For RNAi experiments, the *twisted gastrulation*, *shortened gastrulation*, and *brinker* nucleotide coding sequences were retrieved from each species’ genome annotations [39], and a template DNA fragment for dsRNA synthesis was synthesized into gBlock Gene Fragments (Integrated DNA Technologies, Newark, NJ) (see S2 Table for gBlock and fused T7 promoter-gene primer sequences). Twist dsRNA was synthesized and purified using the MEGAscript T7 and MEGAclear kits, respectively (Invitrogen, Waltham, MA). Developing individuals were reared in artificial brood balls as described above. Once an individual reached the second day of the third instar larval stage, they were injected with either three µg of dsRNA (3 µL of 1 µg/µL dsRNA) or 3 µL of injection buffer as described in ref [60].. Individuals were then returned to their artificial brood balls to complete development. Pictures of beetle head horns or ridges were taken using a digital camera (Scion) mounted on a Leica MZ-16 stereomicroscope. Each time photographs were taken, a reference micrometer image was taken to calibrate length measurements. Horn or ridge length measurements were taken in ImageJ. Curvature measurements were calculated using the *Kappa* plugin [61] in ImageJ. Horn length to body size relationships were compared between control and dsRNA injections via an analysis of covariance (ANCOVA): Horn Length ~ Prothorax Width + Injection Type + Prothorax Width: Injection Type.

## Supporting Information

S1 Fig Repetitive element composition and kimura distances of the *O. sagittarius* and *O. taurus* genomes. LTR: long terminal repeat; SINE: short interspersed nuclear element; LINE: long interspersed nuclear element; DNA: DNA transposon.
(TIF)

S2 Fig UpSet plot illustrating overlap of enriched transcription factor motifs within developing head horn open chromatin regions. Ot: *O. taurus*; Os: *O. sagittarius*; M: male; F: female; H: high nutrition; L: low nutrition.
(TIF)

S3 Fig *brk* RNAi results for *O. taurus* major males. Prothorax width and horn length relationships for control (buffer) and *brk* dsRNA injected individuals (from Fig 4c). P-value from ANCOVA (horn_length ~ prothorax_width + injection + prothorax_width:injection).
(TIF)

S4 Fig Wildtype *O. sagittarius* late-stage female pupae. Developing adult horn sculpted from pupal epithelial layer highlighted on the right.
(TIF)

S5 Fig *brk* and *sog* RNAi results for *O. sagittarius* males. Prothorax width and anterior horn length relationships for control (buffer) and *brk* (left) or *sog* (right) dsRNA injected individuals. P-value from ANCOVA (horn_length ~ prothorax_width + injection + prothorax_width: injection)
(TIF)

S1 Table Differential gene expression of BMP members comparing pupal head horns of major and minor males of *O. taurus.* Green indicates genes with a fold-change > 2 between tissues and supported by a corrected p-value < 0.05. Data from Casasa et al., 2020.
(XLSX)

S2 Table gBlock and primer sequences used for dsRNA synthesis. (XLSX)

S1 Data ATAC-seq accessibility counts (raw and filtered) for *O. taurus* and *O. sagittarius.* (XLSX)

S2 Data Differential expression results of genes nearby horn open chromatin regions. (XLSX)

S3 Data Differential accessibility results for *O. taurus* and *O. sagittarius.* (XLSX)

S4 Data Tests for positive selection results in non-coding genome. (XLSX)

S5 Data Transcription factor motif enrichment and footprinting results. (XLSX)
