# Supplementary material for: The role of gene regulatory evolution in weapon diversification: Insights from comparative genomics, chromatin profiling, and functional analyses of BMP signaling in horned beetles
Source: PLoS Genet. 2026 Oct 21;22(10):e1012338. doi: 10.1371/journal.pgen.1012338 (PMC13705587; doi:10.1371/journal.pgen.1012338)
Supplement: Supplementary file 1 [file pgen.1012338.s013.pdf]

**Response to Reviewers**

**We are pleased to submit a revised version of the manuscript, now titled "The role of gene regulatory evolution in weapon diversification: insights from comparative genomics, chromatin profiling, and functional analyses of BMP signaling in horned beetles." We greatly appreciate the many helpful and constructive comments provided by the reviewers and think that incorporating their suggestions has significantly improved the manuscript. All revisions are addressed point by point below, and we hope that each concern has been adequately addressed. For reference, line numbers refer to the "clean" copy of the manuscript, not the one in which changes are tracked. In brief, we have:**

- **Added a cross-comparison of two RNA-seq datasets with the manuscript's ATAC-seq results**
- **Added an UpSet diagram displaying overlap of transcription factor motif enrichments across conditions/species (S2 Fig) and included a supplemental table describing these results (S5 Data)**
- **Revised our interpretation of the BMP RNA-interference results**
- **Revised language throughout the manuscript to avoid functional assignment of the chromatin accessibility results to developmental phenotype, and more broadly, to accurately describe what conclusions may be drawn from our results and the study's limitations**
- **Provided more precise wording describing the motif analyses**
- **Proposed a revised title and an expanded, more topical description of CRE evolution in the introduction**
- **Incorporated minor clarifications and working changes throughout**

**We would, of course, be happy to consider additional revisions if the reviewers or the editor deems them necessary.**

Reviewer #1: This study investigates the regulatory mechanisms underlying the reshaping of condition-responsive traits in two horned dung beetles, which exhibit highly specialized sex- and nutrition-biased polyphenism. First, the authors assessed intraspecific and interspecific variation in the cis-regulatory landscape using ATAC-seq and identified extensive evolutionary changes in chromatin accessibility under different nutritional and sex conditions. Second, they detected significant transcription factor (TF) motif enrichment of numerous developmental TFs and pathways within putative horn CREs, particularly highlighting the BMP pathway. Finally, they validated the function of conserved BMP pathway in regulating horn development using RNAi and found that the same genes exert pleiotropic effects under different conditions. Overall, these results demonstrate that co-option of highly conserved regulatory networks plays an important role in condition-dependent morphological diversification and provide a clear example of how the pleiotropic effects of a conserved pathway can underlie the same developmental trait across closely related species.
My questions or suggestions for the authors to address as below:
Major comments:
1. Line 170: The authors report that numerous OCRs showing intraspecific differential accessibility and branch-specific positive selection are located near key developmental

regulatory genes, including doublesex, limb expression 1, paired box 6, sox 5, and osa (Fig. 2a). To strengthen the functional relevance of these findings, it would be valuable to assess whether these genes are differentially expressed under different nutritional or sex conditions in O. sagittarius and O. taurus. For example, qRT-PCR analysis could be used to validate potential expression differences.

**Thank you for this suggestion, and we agree that correlating OCR data with expression differences would improve the study and our ability to identify potentially relevant candidate genes. In our revision, we have reanalyzed two RNA-seq datasets in the context of our OCR data that assessed expression differences in the same tissue type: 1) sex differences (Linz et al., 2020; *BMC Biol*) and 2) male nutritional differences (Casasa et al., 2020; *Nat Eco Evol*). Both datasets concern *O. taurus* only – unfortunately, there is no published sex-based RNA-seq dataset for *O. sagittarius*, and male nutritional differences were very modest in Casasa et al. (2020) (only 8 DE genes between high- and low-nutrition males, concordant with this species lacking a horn polyphenism).**

**We then tested whether, at a genome-wide scale, genes with 1 or more differentially accessible (DA) or selection sites nearby were more likely to be differentially expressed than genes with no DA or selection sites nearby. We found no significant difference in these proportions (by Fisher's exact test) in *O taurus*: sex differences → $p > 0.08$; nutrition → $p > 0.88$; selection+sexDE → $p > 0.26$; selection+nutritionDE → $p > 0.76$. For completeness, we asked the same question of genes with 2 or more DA/selection sites nearby, with the same result ($P > 0.20$ in all comparisons). That said, two of the genes we highlight in Fig2a (*dsx* and *lix1)* are differentially expressed between nutritional conditions as well. A complete table of these results is provided in S2 Data and described in the revised text on lines 187-195.**

2. Line 177: The FoxO binding motif is the most enriched in minor males compared with major males or females in O. taurus (Fig. 2e). It would be helpful to clarify whether a similar enrichment pattern is observed in the comparison between females and males in O. sagittarius. In addition, several transcription factor binding motifs, such as Zen.4, ovo.4, and CTCF.2, appear to be consistently enriched under multiple conditions in Fig. 2e. To improve clarity and completeness, I recommend that the authors provide a supplementary table listing all differentially enriched transcription factor binding motifs across conditions. A Venn diagram summarizing shared and condition specific TF binding motifs would further help illustrate the extent of conservation and divergence among conditions.

**Thank you for this helpful suggestion; we agree that providing these results will be more useful to readers. In response, we are now providing an additional table in S5 Data (previously S4 Data) called "motif_overlap.txt", which describes which enriched TF motifs are shared between conditions/species or specific to one set of samples. Because the number of comparisons is relatively high for a Venn diagram, we have also added an UpSet plot to the Supplement (S2 Fig) to illustrate the overlap among significantly enriched motifs, derived from this table. From these additional analyses, we noticed an interesting pattern: hornless-ness, regardless of species or sex, had the most shared enriched motifs (14: *O.***

***sagittarius* males + *O. taurus* females). Sample groups with horn development (*O. sagittarius* females + *O. taurus* males [high and low nutrition]) shared 6 enriched TF motifs. These results are now referenced in the revised text on lines 203-210.**

3. The TF motif enrichment and footprinting analyses identified significantly more zen binding events in head horn OCRs of O. sagittarius males compared with females, as well as in O. taurus major males compared with minor males (Fig. 2b, e). Given this consistent pattern, the manuscript would be strengthened by further investigation of the conserved and divergent roles of zen in regulating horn development across these two species, for example through RNAi-mediated functional assays.

**We appreciate this suggestion and fully agree that a more thorough analysis of the BMP-associated proteins in beetle horn development is a promising direction for future experiments, including downstream TFs (like *zen*) and signal transducers (*mad/med*; re: comments #4 below). That said, we think a complete investigation of this pathway is beyond the scope of this manuscript, whose primary objective is to compare the chromatin landscape and underlying sequence of our two focal species and demonstrate how these analyses may identify new pathways for investigation. We hope we have demonstrated this with the three candidate genes whose activity we investigate and present in Fig 4. In response to this comment, the next one, and Reviewer #2's comments on how to more accurately interpret the BMP findings, we have added the following changes to the manuscript text:**

- **Reservations that some BMP-associated genes analyzed in this study are involved in multiple signaling pathways, and our genomic analyses do not exclusively point to just BMP involvement**
- **Related language changes throughout that these are BMP-*associated* genes, not strictly BMP genes**
- **We retained our comments in the conclusion section that much work remains to fully characterize the role of BMP in beetle horn development and diversification (lines 366-380 in the revision).**

4. Tsg is an extracellular modulator of the BMP signaling pathway and plays complex roles in regulating its activity. To better elucidate the role of BMP signaling in horn development, authors are encouraged to perturb additional components of this pathway, for example through RNAi-mediated knockdown of mad, to suppress its activity and assess the resulting phenotypic effects. This approach would enable evaluation of how identical BMP pathway activity contributes to horn development in different dung beetle species and help clarify its evolutionary role.

**Thank you for this suggestion. Please see our response to #3 above regarding future work on the BMP pathway in beetle horns. We definitely believe the strategy and motivation presented by the reviewer are worth pursuing, but also think that, in the interest of focus**

**and the current manuscript's length, this more extensive analysis of BMP function should be presented elsewhere.**

Minor comments:
1. Line 678: "g" --> "h"
2. Line 683: "i" --> "k"; "j" --> "l"

**Thank you; these are now corrected.**

Reviewer #2: In this paper Davidson et al. use ATAC-seq to characterize the chromatin accessibility profiles of thoracic horns in two dung beetle species, in the contexts of horn plasticity and sexual dimorphism. They then perform series of comparative analyses of different sets of peaks, including looking at overlaps between species and sexes and determining which peaks are conserved across a broader range of beetle species. They also scan sets of these peaks for enrichment of known Drosophila transcription factor binding motifs. Then, in the last part of the paper, they examine various candidate genes that have been implicated in Drosophila BMP signaling, including (1) looking for enrichment of BMP-associated TF binding motifs in ATAC peaks, (2) looking for evidence of selection on a few BMP-associated genes, and (3) using RNAi to examine the function of three candidate BMP-associated genes in horn development.

I enjoyed reading this paper. It was a nice follow up to Davidson & Moczek's 2024 PLoS Genetics paper, and primarily differentiates itself from the previous study by adding ATAC-seq data from a second species (O. sagittarius) and including RNAi knockdowns of three BMP-associated genes. Both of these new analyses produced some nice results that should be of interest to researchers interested in plasticity evo-devo. I think this paper could potentially find a home at PLoS Genetics, although I do have reservations with how the paper is written as a describe below:

• Starting on Line 172 the authors refer to motif prediction as "TF binding sites", and that they are doing comparisons of "differential TF binding". This is highly misleading in the absence of chromatin immunoprecipitation data. Accessibility footprinting is not sufficient to make statements like "we detected 358 CTCF binding events". Throughout the paper and figures the authors must reword all references to "TF binding" to more accurately represent what they really mean: motif enrichment.

**Thank you for raising this concern, and we agree a more precise description of these results would be helpful. We agree that using the terms "TF binding/binding events" to refer to the motif enrichment analyses (Figure 2e, Section 2.3) was inappropriate, as these analyses only searched for motifs of TFs within sets of OCR sequences of interest (i.e., those with evidence of positive selection and differentially accessible between conditions). Each of these occurrences has been corrected in our revised manuscript (binding site/event → binding motif)**

**Section 2.2 also includes ATAC footprinting analyses. Here, we thought the "TF binding" phrasing was more appropriate, given that this terminology is used extensively throughout the ATAC footprinting literature (for example, the method we implement here: TOBIAS-Bentsen et al., 2020; *Nat Comm*). We think that by integrating accessibility and motif enrichment into a single analysis, footprinting does offer a more nuanced prediction of possible TF activity than motif enrichment analyses alone. That said, the point is well taken: footprinting remains a computational prediction and is not a direct way to measure binding events like ChIP. To most accurately describe these results and avoid miscommunication, we have changed all instances of "binding sites/events/scores" to "footprinting sites/events/scores" and emphasized that this analysis is a computational prediction of TF activity rather than a direct measure of it. We hope this change will avoid implying a direct measurement of binding events while distinguishing these analyses from an enrichment analysis of sequence data alone.**

**Similar changes were made to the Author Summary and Abstract, as well.**

**Lastly, appropriate changes to Figure 2 and its legend have been made (Figure 3 already used the correct "motif/motif enrichment" phrasing.**

• Throughout the paper the authors have a habit of overinterpreting their findings, including constantly trying to add evolutionary twists to their interpretations. It became exhausting for me to read sometimes, and I did not try to catalog all of the cases. In the best cases it felt self-conscious and awkward to read, and in the worst cases misleading. Throughout the manuscript the authors should dial back on the overinterpretation and focus more on actual findings. Two examples:

**We thank the reviewer for the candid feedback on the manuscript's text. In our revision, we have made numerous changes throughout to address this concern (please see "tracked changes" version of revised manuscript). The nature of our edits ranges from phrasing/wording changes, for example:**

- **We have removed references to "function/phenotype" from the comparative ATAC and genomic results and clarified that these are "associations/correlations/etc."**
- **Remind readers throughout that ATAC-seq identifies OCRs, which at best identifies the location of *putative* CREs**
- **Made appropriate corrections to "binding site" vs "binding site motif" and BMP pathway members (see below for later comments)**

**To removing or restructuring entire sentences or paragraphs:**

- **Lines 96-104, 179-181, 346-350, to name a few.**

**We address the specific examples raised, as well:**

o "Many of these elements also differ in chromatin accessibility (open vs closed), suggesting that regulation of their activity plays a role in horn morphogenesis." - Simply observing a difference in accessibility is a long way from finding evidence for a role in morphogenesis of a specific trait.

**Previous: Many of these elements also differ in chromatin accessibility (open vs closed), suggesting that regulation of their activity plays a role in horn morphogenesis.**

**Revision (line 44-45): We find that hundreds of elements differ in chromatin accessibility between conditions, including many elements that occur in only a single species.**

**Rather than making a claim about OCR activity and development, we use this space to describe a different finding (presence/absence of lineage-specific OCRs).**

o "These findings suggest that evolutionary changes in chromatin accessibility may play a pivotal role in driving intraspecific diversification of this trait among closely related taxa." – This is misleading without actual connections to phenotypes. OCR profiles are variable between species anywhere you look, and these kinds of differences could simply be due to drift or possibly some other head-related trait.

**Previous: "Although further comparisons across more species are necessary to strengthen this association, these findings suggest that evolutionary changes in chromatin accessibility may play a pivotal role in driving intraspecific diversification of this trait among closely related taxa."**

**Revision (line 146-156): While the correlation between differential accessibility and exaggerated horn phenotypes in either species raises the possibility that chromatin configuration may play a pivotal role in regulating intraspecific variation of this trait, many explanations for these accessibility differences beyond regulation of horn development exist. This is especially pertinent given that chromatin accessibility alone is a permissive rather than instructive feature of transcription factor binding and, by extension, gene regulation (Sexton and Cavalli, 2015). For example, chromatin accessibility differences in horn epithelial cells may have arisen in progenitor cells during an earlier developmental process unrelated to horn morphogenesis or may simply reflect drift between species. Further comparisons across more species and functional studies assessing putative CRE activity will help clarify the relationship between chromatin configuration and changes in horn development.**

• The title doesn't really reflect what's in the paper, and in my opinion is misleading and also vague. The paper does not have much "gene regulatory evolution" content. It is focused on chromatin accessibility profiles, but there is little content related to actual gene regulation. And I don't really see any actual association between the chromatin accessibility, sequence divergence, and "rapid diversification" of beetle horns. The actual chromatin accessibility data are form only two species, and there are no functional data (e.g. reporters or deletions) associating any specific

ATAC peak with beetle horn development. I understand the experimental limitations of the beetle system, but the authors need to be honest about what they can actually infer. I recommend that the authors give the paper a title that more accurately reflects the content of the paper, perhaps mentioning chromatin accessibility profiling of plasticity and sexual dimorphism in beetle horns. This would be very helpful for readers.

**Thank you for these suggestions regarding the title. While we respectfully disagree with the assessment that "the paper does not have much 'gene regulatory evolution' content", we do recognize the need to be more specific. We have therefore revised the title of the manuscript to more precisely reflect the work presented, which now reads: *"The role of gene regulatory evolution in weapon diversification: insights from comparative genomics, chromatin profiling, and functional analyses of BMP signaling in horned beetles".* We hope that this revised title adequately addresses the reviewer's concern.**

• Line 98: "Cis-regulatory elements (CREs) are likely 98 subject to fewer pleiotropic constraints than coding sequences, as their activity is often restricted to specific cell types and developmental stages (Wray et al., 2003; Carroll, 2008; Chan et al., 2009)." These references and ideas are quite old, and this model is challenged in the recent literature (see work by Wittkopp, Stern, etc.). Being open to some more recent viewpoints of regulatory pleiotropy could make the paper feel more topical and also be helpful for interpreting data.

**This is a great point, and we agree a more complete introduction to this topic would be useful to readers. We have moved this sentence to the "Introduction" and added a description of more contemporary work on cis-/trans-regulation and pleiotropy. This updated paragraph is located on lines 80-94 in the revised manuscript.**

• Line 221: A list of TFs "involved in BMP signaling" are presented. This characterization is not totally inaccurate, but the truth is that some of these genes are responsive to multiple signaling pathways (e.g. wingless, dpp, etc.), depending on context, and not just BMP. So calling these "BMP TFs" or "BMP signaling genes", and using enrichment of their binding motifs in OCRs as a sign of "BMP pathway" involvement could be misleading. I would like the authors to be more accurate with their characterization of these elements. (A better way to identify targets of BMP signaling would be to perform chromatin immunoprecipitation with either of the signal transduction TFs, mad or madea.)

**Thank you for raising this concern. In our revision, we now explicitly note that some of the genes examined are not exclusive to the BMP pathway (lines 253-261) and that we cannot rule out additional pathways involving some of these genes. We have also changed the phrasing from "BMP genes" to "BMP-associated genes" to convey a more agnostic interpretation of the results (a hint taken from the introductory paragraph of this review). For our RNAi results, we think it is important to mention that (to our knowledge) *tsg* and *sog* are primary components of BMP (=dpp) signaling, though *brk* does play a role in both BMP (dpp) and Wnt (wg) pathways.**

**Finally, similarly motivated changes to this manuscript's discussion of BMP signaling are included in the Discussion section as part of our response to this review's final comment (see below), the Author Summary, and figure legends.**

• Line 251: "DA" is an undefined abbreviation at this point in the manuscript.

**Corrected.**

• The first paragraph of the Discussion oversells the findings of the paper. "More broadly, these results highlight how phenotypic responses to selection can, over short evolutionary timescales, may proceed along distinct molecular routes (i.e. accessibility or CRE sequence) to potentially modify gene expression and morphogenesis, thereby helping to overcome developmental constraints imposed by pleiotropic interactions." The cis-regulatory aspects of this paper are data from only two species. They are largely a descriptive comparisons of chromatin accessibility. There are no functional data connecting any putative CREs to "phenotypic responses". Also, accessibility and CRE sequences are often functionally connected (e.g. pioneer factor motifs in CREs). And where did the constraint topic come from?

**We appreciate this feedback on the manuscript's Discussion section. In our revised manuscript, we now provide a more careful, focused description of the work's findings and broader implications (lines 337-350, 358-365). We now explicitly mention some of the study's limitations (2 species, genomic-level analyses) and highlight the need for future work to functionally validate candidate CREs and establish a causal basis linking CRE properties, function, and evolution. We maintain that these genomic-level changes detected for OCRs in horn precursor cells (accessibility, mutation, presence/absence of CREs, footprinting/motif analysis) have interesting and potentially meaningful associations with inter- and intraspecific differences in horn development. However, we removed our speculation about whether phenotypic responses proceed via unique (if any) changes in CRE function, and we emphasize that these findings remain genomic (computational) predictions.**
