# Supplementary material for: The role of gene regulatory evolution in weapon diversification: Insights from comparative genomics, chromatin profiling, and functional analyses of BMP signaling in horned beetles
Source: PLoS Genet. 2026 Oct 21;22(10):e1012338. doi: 10.1371/journal.pgen.1012338 (PMC13705587; doi:10.1371/journal.pgen.1012338)
Supplement: Peer review history

# Peer review: Decision Letter 0

**DOI:** 10.1371/journal.pgen.1012338.r001

**Authors:** Paula E. Cohen, Charles G. Danko

15 May 2026

PGENETICS-D-26-00238

Gene regulatory evolution is associated with rapid diversification of beetle horns

PLOS Genetics

Dear Dr. Davidson,

Thank you for submitting your manuscript to PLOS Genetics. After careful consideration, we feel that it has merit but does not fully meet PLOS Genetics's publication criteria as it currently stands. Therefore, we invite you to submit a revised version of the manuscript that addresses the points raised during the review process. We emphasize that it will be important to fully address the reviewer's constructive comments prior to accepting in PLoS Genetics.

Please submit your revised manuscript by Jul 14 2026 11:59PM. If you will need more time than this to complete your revisions, please reply to this message or contact the journal office at plosgenetics@plos.org. When you're ready to submit your revision, log on to https://www.editorialmanager.com/pgenetics/ and select the 'Submissions Needing Revision' folder to locate your manuscript file.

Please include the following items when submitting your revised manuscript:

* A letter that responds to each point raised by the editor and reviewer(s). You should upload this letter as a separate file labeled 'Response to Reviewers'. This file does not need to include responses to any formatting updates and technical items listed in the 'Journal Requirements' section below.

* A marked-up copy of your manuscript that highlights changes made to the original version. You should upload this as a separate file labeled 'Revised Manuscript with Track Changes'.

* An unmarked version of your revised paper without tracked changes. You should upload this as a separate file labeled 'Manuscript'.

If you would like to make changes to your financial disclosure, competing interests statement, or data availability statement, please make these updates within the submission form at the time of resubmission. Guidelines for resubmitting your figure files are available below the reviewer comments at the end of this letter.

As the corresponding author, your ORCID iD is verified in the submission system and will appear in the published article. PLOS supports the use of ORCID, and we encourage all coauthors to register for an ORCID iD and use it as well. Please encourage your coauthors to verify their ORCID iD within the submission system before final acceptance, as unverified ORCID iDs will not appear in the published article. Only the individual author can complete the verification step; PLOS staff cannot verify ORCID iDs on behalf of authors.

We look forward to receiving your revised manuscript.

Kind regards,

Charles G. Danko, Ph.D.

Academic Editor

PLOS Genetics

Paula Cohen

Section Editor

PLOS Genetics

Aimée Dudley

Editor-in-Chief

PLOS Genetics

Anne Goriely

Editor-in-Chief

PLOS Genetics

**Journal Requirements:**

1) Please ensure that the CRediT author contributions listed for every co-author are completed accurately and in full.

At this stage, the following Authors/Authors require contributions: Phillip Davidson, Isabel G. Manley, and Armin P. Moczek. Please ensure that the full contributions of each author are acknowledged in the "Add/Edit/Remove Authors" section of our submission form.

The list of CRediT author contributions may be found here: https://journals.plos.org/plosgenetics/s/authorship#loc-author-contributions

2) Please upload all main figures as separate Figure files in .tif or .eps format. For more information about how to convert and format your figure files please see our guidelines:

https://journals.plos.org/plosgenetics/s/figures

3) We have noticed that you have uploaded Supporting Information files, but you have not included a list of legends. Please add a full list of legends for your Supporting Information files after the references list.

4) Some material included in your submission may be copyrighted. According to PLOS's copyright policy, authors who use figures or other material (e.g., graphics, clipart, maps) from another author or copyright holder must demonstrate or obtain permission to publish this material under the Creative Commons Attribution 4.0 International (CC BY 4.0) License used by PLOS journals. Please closely review the details of PLOS's copyright requirements here: <a data-cke-saved-href="https://journals.plos.org/plosgenetics/s/licenses-and-copyright" href="https://journals.plos.org/plosgenetics/s/licenses-and-copyright">PLOS Licenses and Copyright</a>. If you need to request permissions from a copyright holder, you may use the&nbsp;<a data-cke-saved-href="https://storage.googleapis.com/plos-published-prod/c4ff/Content%20Copyright%20Permission%20Form.pdf?X-Goog-Algorithm=GOOG4-RSA-SHA256&X-Goog-Credential=wombat-sa%40plos-prod.iam.gserviceaccount.com%2F20220817%2Fauto%2Fstorage%2Fgoog4_request&X-Goog-Date=20220817T210608Z&X-Goog-Expires=86400&X-Goog-SignedHeaders=host&X-Goog-Signature=cd9dc868773378f7033b9f8cfb91430167d6275df90bce01342a454f36512530b64f24736fbf68c4fd96da74d663b6ee95b3420bded8fb0e833af2390b896b8706643b476d7f532408921b60576d65b60617070900fd2bcd28eec4cd32fefcf007d3655df17fcc91023329a28479ce0d1c5758174eedcc9e3c1788c5f7dd8523f3ddd871e02c998a20f7dc78ec00f313d4b5a78ba2037a6c7ef0a05dc29817da9368065460c8d8446659f35f0458fefabd3101eb7f926e9cb7a0b53e7bbe47aaed3e0ba52f9af9c2b8f6d7d3a8871b205fc4cbe97384ed37c7604fd7eecfedccf49cba12820b2e3b649541cefde3103cd7d72731b3e8b0248439b4cba691ca8a" href="https://storage.googleapis.com/plos-published-prod/c4ff/Content%20Copyright%20Permission%20Form.pdf?X-Goog-Algorithm=GOOG4-RSA-SHA256&X-Goog-Credential=wombat-sa%40plos-prod.iam.gserviceaccount.com%2F20220817%2Fauto%2Fstorage%2Fgoog4_request&X-Goog-Date=20220817T210608Z&X-Goog-Expires=86400&X-Goog-SignedHeaders=host&X-Goog-Signature=cd9dc868773378f7033b9f8cfb91430167d6275df90bce01342a454f36512530b64f24736fbf68c4fd96da74d663b6ee95b3420bded8fb0e833af2390b896b8706643b476d7f532408921b60576d65b60617070900fd2bcd28eec4cd32fefcf007d3655df17fcc91023329a28479ce0d1c5758174eedcc9e3c1788c5f7dd8523f3ddd871e02c998a20f7dc78ec00f313d4b5a78ba2037a6c7ef0a05dc29817da9368065460c8d8446659f35f0458fefabd3101eb7f926e9cb7a0b53e7bbe47aaed3e0ba52f9af9c2b8f6d7d3a8871b205fc4cbe97384ed37c7604fd7eecfedccf49cba12820b2e3b649541cefde3103cd7d72731b3e8b0248439b4cba691ca8a">PLOS Content Copyright Permission&nbsp;Form</a>

Please respond directly to this email and provide any known details concerning your material's license terms and permissions required for reuse, even if you have not yet obtained copyright permissions or are unsure of your material's copyright compatibility. Once you have responded and addressed all other outstanding technical requirements, you may resubmit your manuscript within Editorial Manager.

Potential Copyright Issues:

- Please confirm (a) that you are the photographer of Figures 1 and 4., or (b) provide written permission from the photographer to publish the photo(s) under our CC BY 4.0 license.

**Reviewers' comments:**

Reviewer's Responses to Questions

**Comments to the Authors:**

**Please note here if the review is uploaded as an attachment.**

Reviewer #1: This study investigates the regulatory mechanisms underlying the reshaping of condition-responsive traits in two horned dung beetles, which exhibit highly specialized sex- and nutrition-biased polyphenism. First, the authors assessed intraspecific and interspecific variation in the cis-regulatory landscape using ATAC-seq and identified extensive evolutionary changes in chromatin accessibility under different nutritional and sex conditions. Second, they detected significant transcription factor (TF) motif enrichment of numerous developmental TFs and pathways within putative horn CREs, particularly highlighting the BMP pathway. Finally, they validated the function of conserved BMP pathway in regulating horn development using RNAi and found that the same genes exert pleiotropic effects under different conditions. Overall, these results demonstrate that co-option of highly conserved regulatory networks plays an important role in condition-dependent morphological diversification and provide a clear example of how the pleiotropic effects of a conserved pathway can underlie the same developmental trait across closely related species.

My questions or suggestions for the authors to address as below:

Major comments:

1. Line 170: The authors report that numerous OCRs showing intraspecific differential accessibility and branch-specific positive selection are located near key developmental regulatory genes, including doublesex, limb expression 1, paired box 6, sox 5, and osa (Fig. 2a). To strengthen the functional relevance of these findings, it would be valuable to assess whether these genes are differentially expressed under different nutritional or sex conditions in O. sagittarius and O. taurus. For example, qRT-PCR analysis could be used to validate potential expression differences.

2. Line 177: The FoxO binding motif is the most enriched in minor males compared with major males or females in O. taurus (Fig. 2e). It would be helpful to clarify whether a similar enrichment pattern is observed in the comparison between females and males in O. sagittarius. In addition, several transcription factor binding motifs, such as Zen.4, ovo.4, and CTCF.2, appear to be consistently enriched under multiple conditions in Fig. 2e. To improve clarity and completeness, I recommend that the authors provide a supplementary table listing all differentially enriched transcription factor binding motifs across conditions. A Venn diagram summarizing shared and condition specific TF binding motifs would further help illustrate the extent of conservation and divergence among conditions.

3. The TF motif enrichment and footprinting analyses identified significantly more zen binding events in head horn OCRs of O. sagittarius males compared with females, as well as in O. taurus major males compared with minor males (Fig. 2b, e). Given this consistent pattern, the manuscript would be strengthened by further investigation of the conserved and divergent roles of zen in regulating horn development across these two species, for example through RNAi-mediated functional assays.

4. Tsg is an extracellular modulator of the BMP signaling pathway and plays complex roles in regulating its activity. To better elucidate the role of BMP signaling in horn development, authors are encouraged to perturb additional components of this pathway, for example through RNAi-mediated knockdown of mad, to suppress its activity and assess the resulting phenotypic effects. This approach would enable evaluation of how identical BMP pathway activity contributes to horn development in different dung beetle species and help clarify its evolutionary role.

Minor comments:

1. Line 678: “g” --> “h”

2. Line 683: “i” --> “k”; “j” --> “l”

Reviewer #2: In this paper Davidson et al. use ATAC-seq to characterize the chromatin accessibility profiles of thoracic horns in two dung beetle species, in the contexts of horn plasticity and sexual dimorphism. They then perform series of comparative analyses of different sets of peaks, including looking at overlaps between species and sexes and determining which peaks are conserved across a broader range of beetle species. They also scan sets of these peaks for enrichment of known Drosophila transcription factor binding motifs. Then, in the last part of the paper, they examine various candidate genes that have been implicated in Drosophila BMP signaling, including (1) looking for enrichment of BMP-associated TF binding motifs in ATAC peaks, (2) looking for evidence of selection on a few BMP-associated genes, and (3) using RNAi to examine the function of three candidate BMP-associated genes in horn development.

I enjoyed reading this paper. It was a nice follow up to Davidson & Moczek’s 2024 PLoS Genetics paper, and primarily differentiates itself from the previous study by adding ATAC-seq data from a second species (O. sagittarius) and including RNAi knockdowns of three BMP-associated genes. Both of these new analyses produced some nice results that should be of interest to researchers interested in plasticity evo-devo. I think this paper could potentially find a home at PLoS Genetics, although I do have reservations with how the paper is written as a describe below:

• Starting on Line 172 the authors refer to motif prediction sas “TF binding sites”, and that they are doing comparisons of “differential TF binding”. This is highly misleading in the absence of chromatin immunoprecipitation data. Accessibility footprinting is not sufficient to make statements like “we detected 358 CTCF binding events”. Throughout the paper and figures the authors must reword all references to “TF binding” to more accurately represent what they really mean: motif enrichment.

• Throughout the paper the authors have a habit of overinterpreting their findings, including constantly trying to add evolutionary twists to their interpretations. It became exhausting for me to read sometimes, and I did not try to catalog all of the cases. In the best cases it felt self-conscious and awkward to read, and in the worst cases misleading. Throughout the manuscript the authors should dial back on the overinterpretation and focus more on actual findings. Two examples:

o “Many of these elements also differ in chromatin accessibility (open vs closed), suggesting that regulation of their activity plays a role in horn morphogenesis.” - Simply observing a difference in accessibility is a long way from finding evidence for a role in morphogenesis of a specific trait.

o “These findings suggest that evolutionary changes in chromatin accessibility may play a pivotal role in driving intraspecific diversification of this trait among closely related taxa.” – This is misleading without actual connections to phenotypes. OCR profiles are variable between species anywhere you look, and these kinds of differences could simply be due to drift or possibly some other head-related trait.

• The title doesn’t really reflect what’s in the paper, and in my opinion is misleading and also vague. The paper does not have much “gene regulatory evolution” content. It is focused on chromatin accessibility profiles, but there is little content related to actual gene regulation. And I don’t really see any actual association between the chromatin accessibility, sequence divergence, and “rapid diversification” of beetle horns. The actual chromatin accessibility data are form only two species, and there are no functional data (e.g. reporters or deletions) associating any specific ATAC peak with beetle horn development. I understand the experimental limitations of the beetle system, but the authors need to be honest about what they can actually infer. I recommend that the authors give the paper a title that more accurately reflects the content of the paper, perhaps mentioning chromatin accessibility profiling of plasticity and sexual dimorphism in beetle horns. This would be very helpful for readers.

• Line 98: “Cis-regulatory elements (CREs) are likely 98 subject to fewer pleiotropic constraints than coding sequences, as their activity is often restricted to specific cell types and developmental stages (Wray et al., 2003; Carroll, 2008; Chan et al., 2009).” These references and ideas are quite old, and this model is challenged in the recent literature (see work by Wittkopp, Stern, etc.). Being open to some more recent viewpoints of regulatory pleiotropy could make the paper feel more topical and also be helpful for interpreting data.

• Line 221: A list of TFs “involved in BMP signaling” are presented. This characterization is not totally inaccurate, but the truth is that some of these genes are responsive to multiple signaling pathways (e.g. wingless, dpp, etc.), depending on context, and not just BMP. So calling these “BMP TFs” or “BMP signaling genes”, and using enrichment of their binding motifs in OCRs as a sign of “BMP pathway” involvement could be misleading. I would like the authors to be more accurate with their characterization of these elements. (A better way to identify targets of BMP signaling would be to perform chromatin immunoprecipitation with either of the signal transduction TFs, mad or madea.)

• Line 251: “DA” is an undefined abbreviation at this point in the manuscript.

• The first paragraph of the Discussion oversells the findings of the paper. “More broadly, these results highlight how phenotypic responses to selection can, over short evolutionary timescales, may proceed along distinct molecular routes (i.e. accessibility or CRE sequence) to potentially modify gene expression and morphogenesis, thereby helping to overcome developmental constraints imposed by pleiotropic interactions.” The cis-regulatory aspects of this paper are data from only two species. They are largely a descriptive comparisons of chromatin accessibility. There are no functional data connecting any putative CREs to “phenotypic responses”. Also, accessibility and CRE sequences are often functionally connected (e.g. pioneer factor motifs in CREs). And where did the constraint topic come from?

**********

**Have all data underlying the figures and results presented in the manuscript been provided?**

Large-scale datasets should be made available via a public repository as described in the *PLOS Genetics*  data availability policy, and numerical data that underlies graphs or summary statistics should be provided in spreadsheet form as supporting information.

Reviewer #1: Yes

Reviewer #2: Yes

**********

PLOS authors have the option to publish the peer review history of their article (what does this mean?). If published, this will include your full peer review and any attached files.

If you choose “no”, your identity will remain anonymous but your review may still be made public.

**Do you want your identity to be public for this peer review?**  For information about this choice, including consent withdrawal, please see our Privacy Policy.

Reviewer #1: No

Reviewer #2: No

[NOTE: If reviewer comments were submitted as an attachment file, they will be attached to this email and accessible via the submission site. Please log into your account, locate the manuscript record, and check for the action link "View Attachments". If this link does not appear, there are no attachment files.]

**Figure resubmission:**

**Reproducibility:**

To enhance the reproducibility of your results, we recommend that authors of applicable studies deposit laboratory protocols in protocols.io, where a protocol can be assigned its own identifier (DOI) such that it can be cited independently in the future. Additionally, PLOS ONE offers an option to publish peer-reviewed clinical study protocols. Read more information on sharing protocols at https://plos.org/protocols?utm_medium=editorial-email&utm_source=authorletters&utm_campaign=protocols

# Author comment: Author response to Decision Letter 1

**DOI:** 10.1371/journal.pgen.1012338.r002

5 Aug 2026

Attachment Submitted filename: Response to Reviewers.pdf

# Peer review: Decision Letter 1

**DOI:** 10.1371/journal.pgen.1012338.r003

**Authors:** Paula E. Cohen, Charles G. Danko

30 Sep 2026

Dear Dr Davidson,

We are pleased to inform you that your manuscript entitled "The role of gene regulatory evolution in weapon diversification: insights from comparative genomics, chromatin profiling, and functional analyses of BMP signaling in horned beetles" has been editorially accepted for publication in PLOS Genetics. Congratulations!

Before your submission can be formally accepted and sent to production you will need to complete our formatting changes, which you will receive in a follow up email. Please be aware that it may take several days for you to receive this email; during this time no action is required by you. Please note: the accept date on your published article will reflect the date of this provisional acceptance, but your manuscript will not be scheduled for publication until the required changes have been made.

Once your paper is formally accepted, an uncorrected proof of your manuscript will be published online ahead of the final version, unless you’ve already opted out via the online submission form. If, for any reason, you do not want an earlier version of your manuscript published online or are unsure if you have already indicated as such, please let the journal staff know immediately at plosgenetics@plos.org.

In the meantime, please log into Editorial Manager at https://www.editorialmanager.com/pgenetics/, click the "Update My Information" link at the top of the page, and update your user information to ensure an efficient production and billing process. Note that PLOS requires an ORCID iD for all corresponding authors. Therefore, please ensure that you have an ORCID iD and that it is validated in Editorial Manager. To do this, go to ‘Update my Information’ (in the upper left-hand corner of the main menu), and click on the Fetch/Validate link next to the ORCID field.  This will take you to the ORCID site and allow you to create a new iD or authenticate a pre-existing iD in Editorial Manager.

If you have a press-related query, or would like to know about making your underlying data available (as you will be aware, this is required for publication), please see the end of this email. If your institution or institutions have a press office, please notify them about your upcoming article at this point, to enable them to help maximise its impact. Inform journal staff as soon as possible if you are preparing a press release for your article and need a publication date.

Thank you again for supporting open-access publishing; we are looking forward to publishing your work in PLOS Genetics!

Yours sincerely,

Charles G. Danko, Ph.D.

Academic Editor

PLOS Genetics

Marnie Blewitt

Section Editor

PLOS Genetics

Aimée Dudley

Editor-in-Chief

PLOS Genetics

Anne Goriely

Editor-in-Chief

PLOS Genetics

www.plosgenetics.org

BlueSky: @plos.bsky.social

----------------------------------------------------

Comments from the reviewers (if applicable):

Reviewer's Responses to Questions

**Comments to the Authors:**

**Please note here if the review is uploaded as an attachment.**

Reviewer #1: All my concerns have been resolved.

Reviewer #2: I have read the revised manuscript and I believe it is acceptable for publication. I think the authors for their thoughtful responses and revisions.

**********

**Have all data underlying the figures and results presented in the manuscript been provided?**

Large-scale datasets should be made available via a public repository as described in the *PLOS Genetics*  data availability policy, and numerical data that underlies graphs or summary statistics should be provided in spreadsheet form as supporting information.

Reviewer #1: Yes

Reviewer #2: Yes

**********

PLOS authors have the option to publish the peer review history of their article (what does this mean?). If published, this will include your full peer review and any attached files.

If you choose “no”, your identity will remain anonymous but your review may still be made public.

**Do you want your identity to be public for this peer review?**  For information about this choice, including consent withdrawal, please see our Privacy Policy.

Reviewer #1: No

Reviewer #2: No

----------------------------------------------------

**Data Deposition**

If you have submitted a Research Article or Front Matter that has associated data that are not suitable for deposition in a subject-specific public repository (such as GenBank or ArrayExpress), one way to make that data available is to deposit it in the Dryad Digital Repository. As you may recall, we ask all authors to agree to make data available; this is one way to achieve that. A full list of recommended repositories can be found on our website.

The following link will take you to the Dryad record for your article, so you won't have to re‐enter its bibliographic information, and can upload your files directly:

http://datadryad.org/submit?journalID=pgenetics&manu=PGENETICS-D-26-00238R1

More information about depositing data in Dryad is available at http://www.datadryad.org/depositing. If you experience any difficulties in submitting your data, please contact help@datadryad.org for support.

Additionally, please be aware that our data availability policy requires that all numerical data underlying display items are included with the submission, and you will need to provide this before we can formally accept your manuscript, if not already present.

----------------------------------------------------

**Press Queries**

If you or your institution will be preparing press materials for this manuscript, or if you need to know your paper's publication date for media purposes, please inform the journal staff as soon as possible so that your submission can be scheduled accordingly. Your manuscript will remain under a strict press embargo until the publication date and time. This means an early version of your manuscript will not be published ahead of your final version. PLOS Genetics may also choose to issue a press release for your article. If there's anything the journal should know or you'd like more information, please get in touch via plosgenetics@plos.org.

# Editor report: Acceptance letter

**DOI:** 10.1371/journal.pgen.1012338.r004

**Authors:** Paula E. Cohen, Charles G. Danko

PGENETICS-D-26-00238R1

The role of gene regulatory evolution in weapon diversification: insights from comparative genomics, chromatin profiling, and functional analyses of BMP signaling in horned beetles

Dear Dr Davidson,

We are pleased to inform you that your manuscript entitled "The role of gene regulatory evolution in weapon diversification: insights from comparative genomics, chromatin profiling, and functional analyses of BMP signaling in horned beetles" has been formally accepted for publication in PLOS Genetics! Your manuscript is now with our production department and you will be notified of the publication date in due course.

The corresponding author will soon be receiving a typeset proof for review, to ensure errors have not been introduced during production. Please review the PDF proof of your manuscript carefully, as this is the last chance to correct any errors. Please note that major changes, or those which affect the scientific understanding of the work, will likely cause delays to the publication date of your manuscript.

Soon after your final files are uploaded, unless you have opted out or your manuscript is a front-matter piece, the early version of your manuscript will be published online. The date of the early version will be your article's publication date. The final article will be published to the same URL, and all versions of the paper will be accessible to readers.

For Research Articles, you will receive an invoice from PLOS for your publication fee after your manuscript has reached the completed accept phase. If you receive an email requesting payment before acceptance or for any other service, this may be a phishing scheme. Learn how to identify phishing emails and protect your accounts at https://explore.plos.org/phishing.

Thank you again for supporting PLOS Genetics and open-access publishing. We are looking forward to publishing your work!

With kind regards,

Sharmila Kamatchi

PLOS Genetics

On behalf of:

The PLOS Genetics Team

Carlyle House, Carlyle Road, Cambridge CB4 3DN | United Kingdom

plosgenetics@plos.org | +44 (0) 1223-442823

plosgenetics.org | Twitter: @PLOSGenetics
